# One-Step Synthesis of Sulfur-Doped Nanoporous Carbons from Lignin with Ultra-High Surface Area, Sulfur Content and CO_2_ Adsorption Capacity

**DOI:** 10.3390/ma16010455

**Published:** 2023-01-03

**Authors:** Dipendu Saha, Gerassimos Orkoulas, Dean Bates

**Affiliations:** Chemical Engineering Department, Widener University, 1 University Place, Chester, PA 19103, USA

**Keywords:** porous carbon, sustainability, surface area, CO_2_ separation

## Abstract

Lignin is the second-most available biopolymer in nature. In this work, lignin was employed as the carbon precursor for the one-step synthesis of sulfur-doped nanoporous carbons. Sulfur-doped nanoporous carbons have several applications in scientific and technological sectors. In order to synthesize sulfur-doped nanoporous carbons from lignin, sodium thiosulfate was employed as a sulfurizing agent and potassium hydroxide as the activating agent to create porosity. The resultant carbons were characterized by pore textural properties, X-ray photoelectron spectroscopy (XPS), X-ray diffraction (XRD), and scanning electron microscopy (SEM). The nanoporous carbons possess BET surface areas of 741–3626 m^2^/g and a total pore volume of 0.5–1.74 cm^3^/g. The BET surface area of the carbon was one of the highest that was reported for any carbon-based materials. The sulfur contents of the carbons are 1–12.6 at.%, and the key functionalities include S=C, S-C=O, and SO_x_. The adsorption isotherms of three gases, CO_2_, CH_4_, and N_2_, were measured at 298 K, with pressure up to 1 bar. In all the carbons, the adsorbed amount was highest for CO_2_, followed by CH_4_ and N_2_. The equilibrium uptake capacity for CO_2_ was as high as ~11 mmol/g at 298 K and 760 torr, which is likely the highest among all the porous carbon-based materials reported so far. Ideally adsorbed solution theory (IAST) was employed to calculate the selectivity for CO_2_/N_2_, CO_2_/CH_4_, and CH_4_/N_2_, and some of the carbons reported a very high selectivity value. The overall results suggest that these carbons can potentially be used for gas separation purposes.

## 1. Introduction

Sulfur-doped porous carbon is a unique form of heteroatom doped carbon. Unlike other common types of heteroatoms, such as nitrogen, oxygen, or boron, sulfur atoms are significantly larger than carbon atoms, and therefore, sulfur atoms protrude out of the graphene plant, giving rise to a few unique properties of the parent carbon, such as superconductivity, as revealed in the theoretical studies [1,2]. In addition, the lone pair of electrons in the sulfur atom induces polarizability and interactions with oxygen [3]. There are several specific applications of sulfur-doped porous carbon, including electrocatalysis for fuel cells [4], electrodes for electrochemical capacitors [5], anode material for Li-ion batteries [6], cathodes for Li-S batteries [7], heavy metal removal [8], toxic gas removal [9], H_2_ storage [10], CO_2_ separation [11], and many others [12]. 

Most of the time, sulfur-doped carbons are synthesized by carbonizing S-bearing carbon precursors, like thiophenemethanol [13], cysteine [14], algae [15], ionic liquids [16], and others [12]. The detailed list of precursors that have been employed to synthesize sulfur-doped nanoporous carbons are listed in [12]. The porosity within the sulfur-doped carbon is achieved by post-synthesis activation [17] or utilizing templating strategies [13], including hard and soft templates. In our past research, we incorporated sodium thiosulfate (Na_2_S_2_O_3_) at elevated temperatures to introduce sulfur functionalities within the porous carbon [8,18,19,20,21,22]. The uniqueness of incorporating Na_2_S_2_O_3_ is that it does not require an S-bearing carbon precursor to synthesize sulfur-doped carbon, as sulfur is contributed by the Na_2_S_2_O_3_.

Lignin is the second most naturally abundant biopolymer present in the environment. It is one of the key constituents of wood along with cellulose and hemicellulose. Although there are three key structural constituents of lignin, including coumaryl, guaiacyl, and sinapyl alcohol, these three components are randomly cross-linked with each other, giving rise to the structural heterogeneity of lignin polymer. The exact structure of lignin polymer depends on the wood (tree) type and processing conditions. Lignin is industrially produced as a by-product in pulp and paper industries and bio-refineries. Although there is much research on the use of lignin, it still lacks prominent value-added utilization. The majority of lignin is used as low-calorie fuel. Historically, lignin was used in several types of specialty carbons, including activated carbon [23,24], mesoporous carbon [25,26], and carbon fibers [27,28]. Synthesis of porous carbon from lignin not may only introduce sustainability in the synthesis but also influences the economy of lignin by increasing its value-added utilization. 

In this work, we synthesized sulfur-doped nanoporous carbon from lignin using a one-step approach. We incorporated a varying ratio of sodium thiosulfate (Na_2_S_2_O_3_) and potassium hydroxide (KOH) to simultaneously introduce sulfur functionalities and porosity into the carbon matrix. The resultant carbon was employed for gas separation purposes. 

## 2. Experimental

### 2.1. Synthesis of Sulfur-Doped Carbons

**Figure 1:**
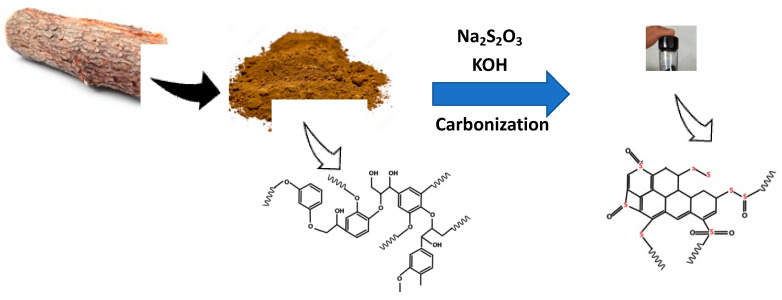
Schematic of one-step synthesis of sulfur-doped nanoporous carbon from lignin.

For all the synthesis purposes, commercially available dealkaline lignin (TCI America) was employed. Typically, the desired components of lignin, sodium thiosulfate (Na_2_S_2_O_3_), and potassium hydroxide (KOH) were mixed in a coffee grinder and then loaded onto an alumina boat. The boat was introduced to the Lindberg-Blue^TM^ (USA) tube furnace. The tube furnace was heated to 800 °C with a ramp rate of 10 °C/min, dwelled at 800 °C for 2 min, and then cooled to room temperature. All the heating and cooling operations were performed under N_2_ gas. The final products were washed several times with DI water and then filtered and dried. The names of the carbons according to the ratio of lignin, Na_2_S_2_O_3_, and KOH are given in Table 1. The schematic of the synthesis is shown in Figure 1. From the table, it is clear that the total mixture was in the range of 7–12 g, which is the maximum amount of materials that be processed within the porcelain boat onto the tube surface. The ratio of Na_2_S_2_O_3_ and KOH was also adjusted according to the literature and our previous fundings; too low or too high amounts of these materials may result in improper impregnation/activation or breakdown of the entire carbon matrix.

### 2.2. Characterization of Sulfur-Doped Carbons

All the carbons were characterized with pore textural properties, x-ray photoelectron spectroscopy (XPS), and scanning electron microscopy (SEM). The pore textural properties, including BET specific surface area (BET SSA) and pore size distribution, were calculated using N_2_ adsorption isotherms at 77 K and CO_2_ adsorption isotherms at 273 K in Quantachorme’s Autosorb iQ-any gas instrument (USA). The non-local density function theory (NLDFT)-based pore size distribution below 12 Å was calculated using CO_2_ adsorption isotherm, whereas the larger (>12 Å) pores were calculated using N_2_ adsorption isotherm. X-ray photoelectron spectroscopy (XPS) results were obtained in a Thermo-Fisher K-alpha instrument (USA)with a monochromatic Al-Kα as an X-ray anode. The intensity of X-ray energy was set to 1486.6 eV, and the resolution was 0.5 eV. Scanning electron microscopic images (SEM) were obtained in a Carl Zeiss Merlin SEM microscope (USA) operating at 1 kV. X-ray diffraction patterns were obtained in Rigaku miniflex XRD instrument. In order to capture the XRD pattern of the carbon, it was ground to a fine powder in mortar and pestle and introduced within the sample holder.

### 2.3. Gas Adsorption Studies

Equilibrium adsorption isotherms of pure-component CO_2_, CH_4_, and N_2_ were measured on all the nanoporous carbons at the temperature of 298 K and pressure up to 760 torr in the same Autosorb iQ-any gas instrument. The temperature was maintained by an additional Chiller (Julabo) (USA). All the gases were of ultra-high purity (UHP) grade. About 80 mg of each of the sample was inserted in the sample tube along with filler rod and non-elutriation cap. Each sample was outgassed at 300 °C for 3 h before the adsorption experiment. 

## 3. Results and Discussion

### 3.1. Material Characteristics

**Figure 2:**
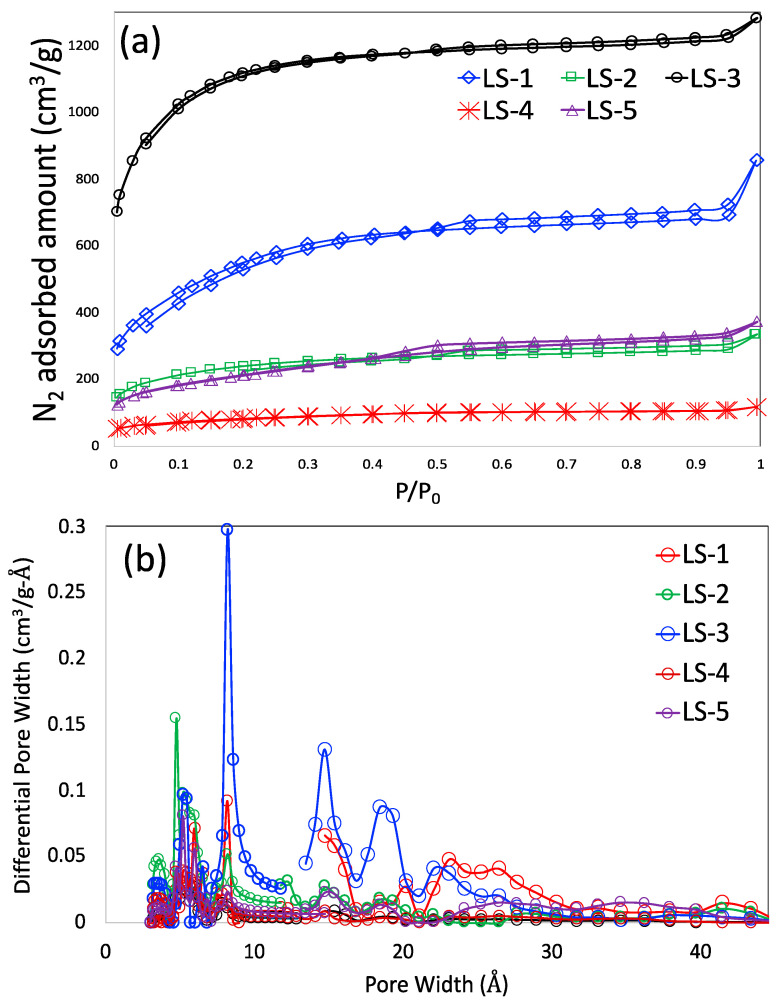
N_2_ adsorption–desorption isotherms at 77 K (**a**); NLDFT-based pore size distribution (**b**).

The N_2_ adsorption-desorption isotherms at 77 K are shown in Figure 2a. The sharp rise in the low-pressure region suggests the presence of macroporosity. A narrow stretch of hysteresis loop is also observed in all the isotherms, signifying the presence of mesoporosity. The NLDFT-based pore size distribution is shown in Figure 2b. This figure shows that all the carbons have a few pores in the narrow micropore region, including 8.1, 5.5, and 4.7 Å; the pore width around 3.4 Å is attributed to the graphite layer spacing and not a true pore. In the large micropore region, the carbons possess two distinct pores around the 14.7 and 19.3 Å regions. The majority of the carbons also demonstrated a distributed mesoporosity within 20–45 Å, supporting the presence of a hysteresis loop in Figure 2a. The detailed pore textural properties are shown in Table 2. It is observed that LS-3 has the highest BET SSA (3626 m^2^/g) and pore volume (1.74 cm^3^/g). A porous carbon with a BET surface area more than 3000 m^2^/g is very difficult to produce and has been rarely reported in the literature [29,30,31,32,33,34]; only one work reported the BET surface area higher than that of LS-3 (a MOF-derived porous carbon with BET: 4300 m^2^/g) [34]. The lowest porosity belongs to LS-4 (BET: 280 m^2^/g; pore volume: 0.157 cm^3^/g), synthesized without KOH. It is clear that KOH is the primary agent in creating the porosity, Na_2_S_2_O_3_ is primarily used to introduce sulfur functionalities. The influence of Na_2_S_2_O_3_ in creating porosity is very small. 

**Figure 3:**
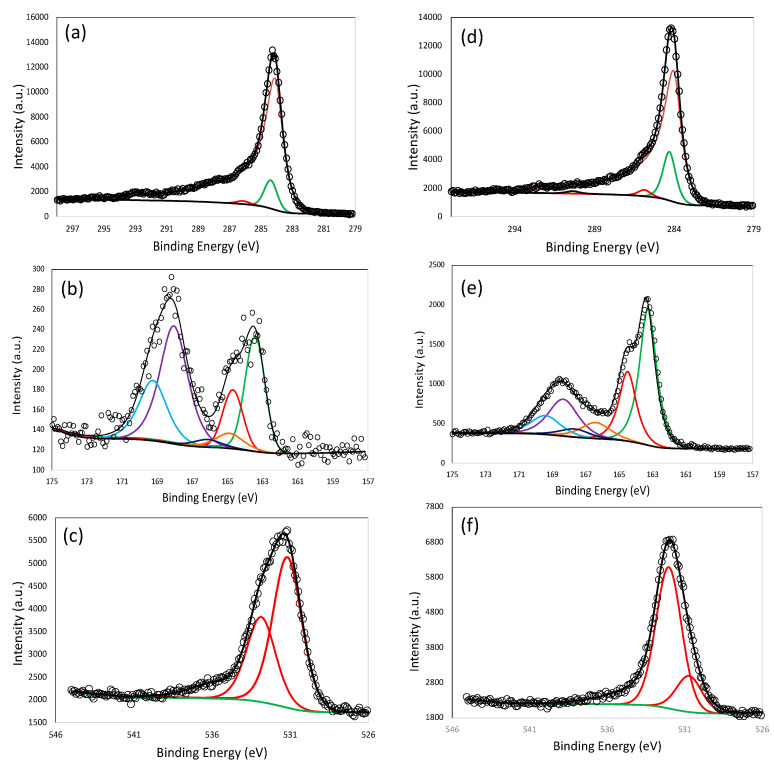
X-ray photoelectron spectroscopy (XPS) analysis; peak deconvolution results of C-1s (**a**), S-2p (**b**), and O-1s (**c**) of LS-3; and C-1s (**d**), S-2p (**e**), and O-1s (**f**) of LS-5.

The quantitative results for XPS are shown in Table 3. The C, S, and O contents were calculated by fitting the C-1s, S-2p, and O-1s peaks, and the representative peak fitting results for LS-3 and LS-5 are shown in Figure 3a–f. As observed in the table, LS-4 has the highest amount of sulfur content (12.6 at.%), followed by LS-5 (8.9 at.%). It is quite intuitive to note that the sulfur content is directly proportional to the addition of Na_2_S_2_O_3_ in the course of synthesis; sulfur content decreases in the order of LS-4 > LS-5 > LS-2 > LS-1 > LS-3. Despite LS-5 and LS-4 having the same Na_2_S_2_O_3_ contents, a higher KOH in LS-5 causes removal of some of the sulfur contents in the course of activation. It is also interesting to note that LS-4 has about 1 at.% sulfur, which originated from the pristine lignin itself in the course of its industrial production. Within different types of sulfur functionalities, the largest fraction of sulfur is associated with C-S contents in all the porous carbon samples, followed by SO_x_ and S=C-O. From Table 3, it is obvious that higher sulfur content also caused higher oxygen content (except LS-3), which might have affected sulfur functionalities, resulting in lowering of total carbon content. Within the oxygen-bearing functionalities, the largest group belonged to S=O/C=O/O-H, directly correlating the oxygen contents with sulfur. LS-4, which had the highest sulfur content, had the lowest total carbon content, only 51.3 at.%. According to XPS, C-C sp^2^ is the primary carbon structure, suggesting that all the carbons are mostly graphitic in nature. It also needs to be noted that the LS-3, LS-4, and LS-5 have sodium that may have originated from Na_2_S_2_O_3_ and/or during the commercial production of lignin.

**Figure 4:**
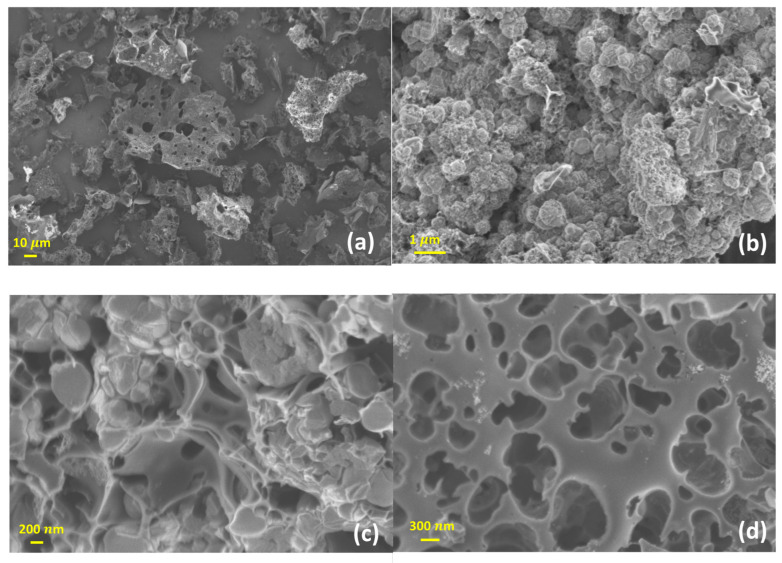
Representative Scanning Electron Imaging (SEM) results of LS-3 (**a**–**d**).

The representative SEM images of LS-3 are shown in Figure 4a–d at different levels of magnification. The carbon particles are irregular in shape, with a size around 20–100 μm. A three-dimensional network of larger pores is observed in the SEM images with macropore size in the range of 1.5–4 μm. Such a pore system along with meso- and micropores may have created a hierarchical porous network in the carbon matrix, which can be highly beneficial for faster diffusion of an adsorbate molecule in the course of diffusion. 

**Figure 5:**
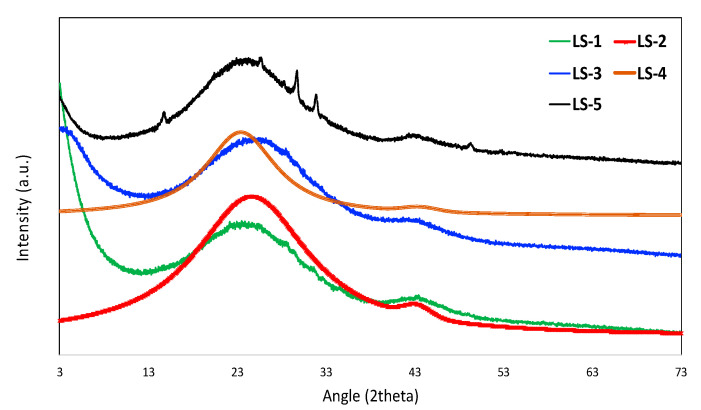
X-ray diffraction (XRD) results.

The X-ray diffraction (XRD) images are shown in Figure 5. Two ‘hump’-like and very broad peaks around 23° and 43° are observed for all the carbons. These peaks are remnants of graphitic ordering and are present in almost all sp^2^ hybridized carbons [35]. For LS-5, there are a few small peaks observed, which may be associated with salts of Na and/or K, which could originate from Na_2_S_2_O_3_, KOH, and the impurities present in pristine lignin itself. Relatively higher amounts of Na also support the XPS observation, and such sodium originates from sodium thiosulfate and/or pristine lignin. We did not pursue any further analysis to reveal details of these salts, as it is beyond the scope of this study. 

### 3.2. Gas Adsorption Studies

**Figure 6:**
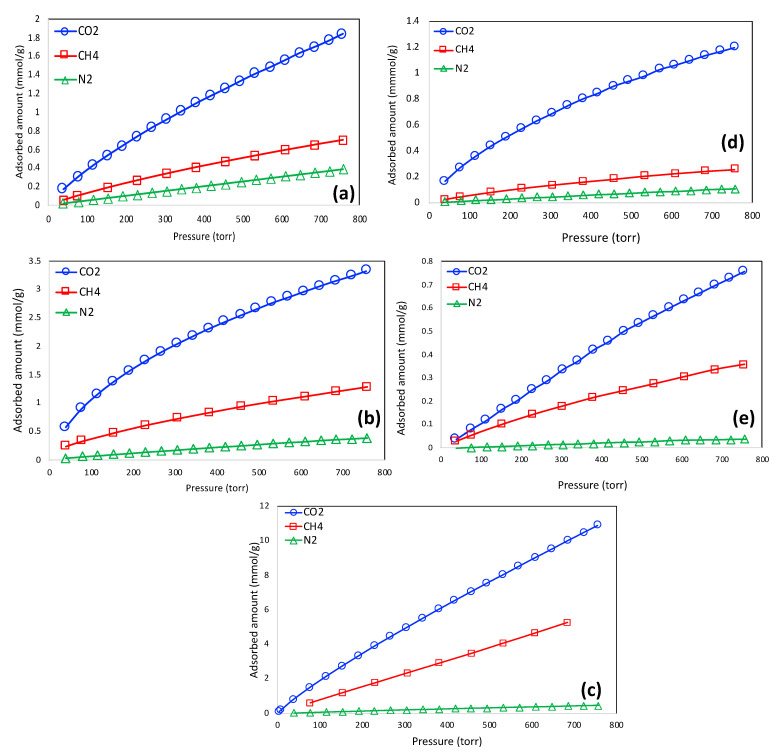
Adsorption isotherms of CO_2_, CH_4_, and N_2_ for LS-1 (**a**), LS-2 (**b**), LS-3 (**c**), LS-4 (**d**), and LS-5 (**e**).

The adsorption isotherms CO_2_, CH_4_, and N_2_ are shown in Figure 6a–e for LS-1, -2, -3, -4, and -5, respectively. For all the plots, the CO_2_ adsorption amount is higher, followed by CH_4_ and N_2_. The largest equilibrium adsorption capacity of CO_2_ was demonstrated by LS-3 (~10.89 mmol/g at 757 torr pressure), which has the highest BET surface area and micropore volume. Such a high equilibrium uptake of CO_2_ is probably the highest CO_2_ uptake capacity ever reported for any carbon-based material in the literature. The adsorption of all these gases is influenced by micropore volume. As observed in this study, there is a linear trend of adsorbed amounts of CO_2_, CH_4_, and N_2_, suggesting that the micropore volume played a pivotal role in the adsorption processes. In addition, CO_2_ adsorption may also be influenced by the presence of sulfur functionalities. It has been reported that mono- or dioxidized sulfur on the carbon surface causes high enthalpy of CO_2_ adsorption of 4–6 kcal/mol, which may be attributed to the negative charge of an oxygen atom, possibly caused by the high positive charge on the sulfur atom [36]. Theoretical calculations also revealed that electron overlap between CO_2_ and sulfur functionalities on the carbon surface may enhance the interactions between CO_2_ and the carbon substrate [37]. As observed in Figure 5, there is a possible presence of Na and K salts, which might have originated from Na_2_S_2_O_3_, KOH, or the pristine lignin itself. To the best of our knowledge, these salts do not have any influence in the adsorption of CO_2_, CH_4_, or N_2_. 

**Figure 7:**
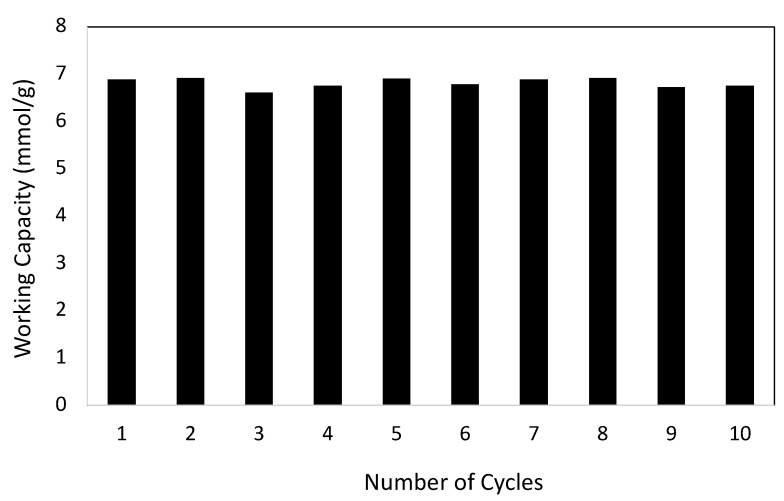
Cyclability of working capacity of CO_2_ adsorption in LS-5.

Working capacity is generally defined as the difference in the adsorbed amount within the adsorbed pressure of 1 bar (760 torr) and desorbed pressure of 0.1 bar (76 torr). For a suitable adsorbent, consistent working capacity with multiple cycles is required. In this work, we have selected LS-5 as the adsorbent and CO_2_ adsorbate gas to study the cyclability of working capacity, and the result is shown in Figure 7. As observed in this figure, the working capacity maintains a constant value within 10 cycles, with a standard deviation of no more than ±0.1.

The gas adsorption isotherms were fitted with the Sips isotherm model equation, given below.

(1) $$q=\frac{a_{m}bp^{1/n}}{1+bp^{1/n}}$$

where q is the adsorbed amount (mmol/g), p is the pressure (torr), and $a_{m}$, b, and n are all Sips constants. The Sips equation is fit employing the solver function of Microsoft Excel, and the values are given in Table S1 of the supporting information. 

**Figure 8:**
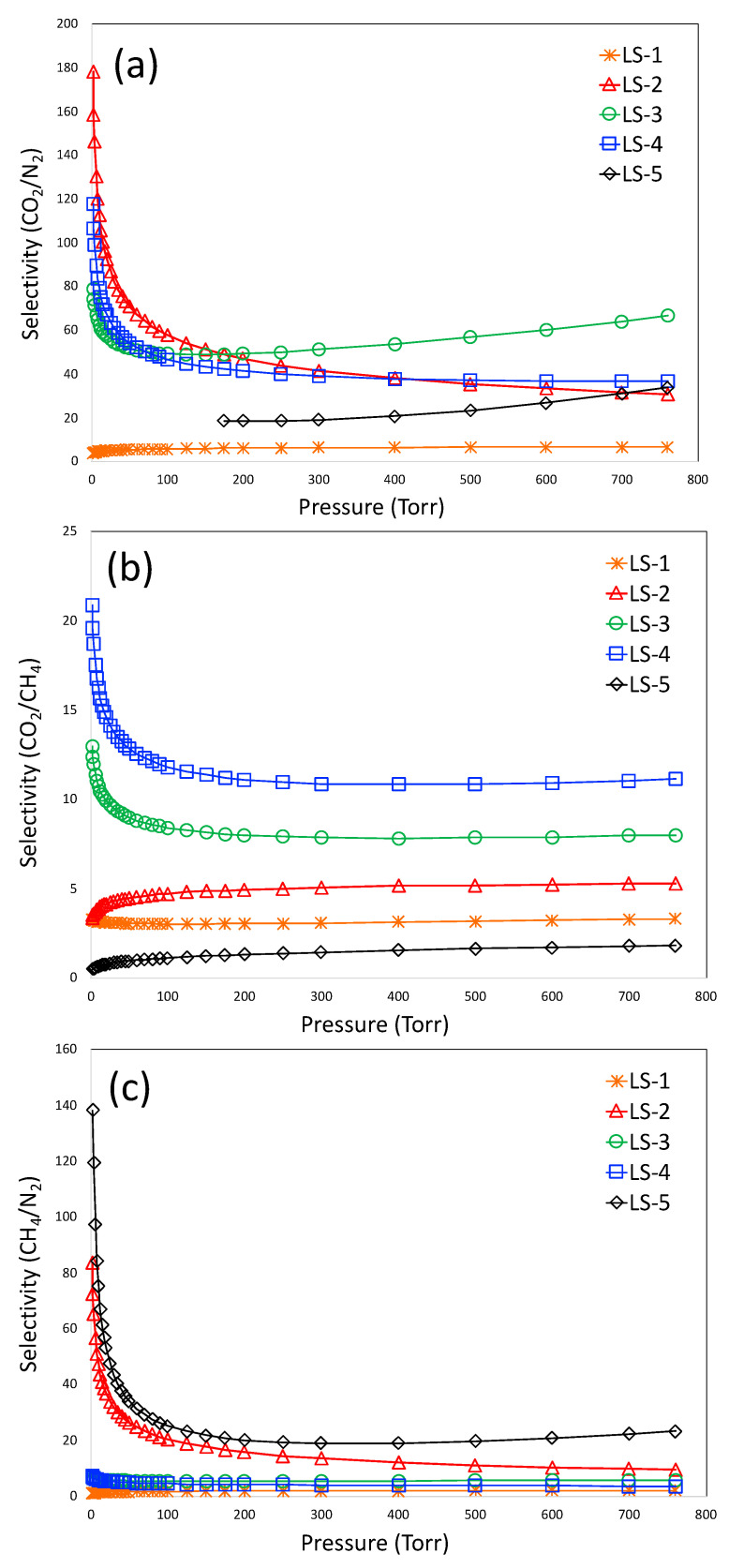
IAST-based selectivity for CO_2_/N_2_ (**a**), CO_2_/CH_4_ (**b**), and (**c**) CH_4_/N_2_.

Owing to the experimental difficulty in performing mixed gas adsorption, it is common practice to calculate the selectivity from the pure-component gas adsorption isotherms. Selectivity provides an indication of the preference of the adsorbent materials to prefer one component over another when both species are present in the feed stream. The selectivity ($\alpha_{1/2}$) of component 1 (preferred adsorbate) over component 2 (non-preferred adsorbate) is defined as follows [38]:

(2) $$\alpha_{1/2}=\frac{x_{1}/y_{1}}{x_{2}/y_{2}}$$

where x and y are the mole fractions of adsorbate in the adsorbed phase and bulk gas phase, respectively. The most popular way of calculating selectivity from adsorption isotherms is the Ideally Adsorbed Solution Theory (IAST), originally proposed by Myers and Prausnitz [39]. The selectivity values for CO_2_/N_2_, CO_2_/CH_4_, and CH_4_/N_2_ are shown in Figure 8a–c, respectively. From Figure 8a, it is observed that LS-2 and LS-4 have the highest selectivity for CO_2_/N_2_ (about 180–120) at the lowest pressure, but it decreases significantly at the higher pressure. At the higher pressure, the highest selectivity was demonstrated by LS-3, which is about 80-60. The lowest selectivity was demonstrated by LS-1. For the selectivity of CO_2_/CH_4_ (Figure 8b), the highest selectivity was demonstrated by LS-4 (20-11), followed by LS-3, LS-2, LS-1, and LS-5. For CH_4_/N_2_, the highest selectivity was demonstrated by LS-2 and LS-5 (Figure 8c), which was about 80–140 in the lower pressure range and 23-9 in the lower pressure range. The selectivity of CO_2_/N_2_ is probably one of the highest among other S-doped porous carbons reported in the literature; only the sulfur-doped mesoporous carbon synthesized from resorcinol-formaldehyde in our previous work [22] demonstrated slightly higher selectivity of 190 compared to that of LS-3 and LS-4. For CO_2_/CH_4_, the selectivity values lie within 3.3–15.7 in the literature [40]. The selectivity of CO_2_/CH_4_ for LS-4 (21-11) is higher than that reported in the literature. The selectivity of CH_4_/N_2_ was reported to be as high as 14 in the literature; LS-2 and LS-5 demonstrated a much higher selectivity than this value. It is also important to note that the high equilibrium uptake capacity of a pure preferred component does not always confirm its high selectivity over a non-preferred component; the selectivity also depends on the shape of the pure-component isotherms of both the preferred and non-preferred component. As an example, LS-5 demonstrated very high equilibrium uptake capacity for CO_2_ and CH_4_; however, it represents the highest selectivity owing to the linear nature of the isotherms. 

## 4. Conclusions

In this work, we successfully synthesized sulfur-doped nanoporous carbon with ultrahigh surface area from lignin by one-step carbonization with the help sodium thiosulfate as a sulfurizing agent and potassium hydroxide as an activating agent. The peak deconvolution results of XPS confirmed that the nanoporous carbons possess the sulfur contents of 1 to 12.6 at.%. The porosity analysis revealed that the BET specific surface areas of the carbons are in the range of 741–3626 m^2^/g. The surface area of 3626 m^2^/g is one of the highest for carbon-based materials reported in literature. Pure-component adsorption isotherms of CO_2_, CH_4_, and N_2_ were measured on all the porous carbons at 298 K, with pressure up to 760 torr. The carbon with the highest BET surface area demonstrated the highest CO_2_ uptake of more than 10.89 mmol/g, which is one of the highest for porous carbon-based materials reported in literature. The IAST method was applied to calculate the selectivity of CO_2_/N_2_, CO_2_/CH_4_, and CH_4_/N_2_ from the pure-component isotherm data, and the results demonstrated that these materials can potentially be used for gas separation purposes.

## Figures and Tables

**Table 1:** Sample identity.

Sample Identity	Lignin: Na_2_S_2_O_3_: KOH
LS-1	3:2:2
LS-2	3:3:1
LS-3	3:1:3
LS-4	3:6:0
LS-5	3:6:3

**Table 2:** Pore textual properties.

Sample Identity	BET SSA (m^2^/g)	Total Pore Volume (cm^3^/g)	Micropore Volume (cm^3^/g)	Mesopore Volume (cm^3^/g)
LS-1	1915	1.079	0.532	0.547
LS-2	787	0.443	0.292	0.151
LS-3	3626	1.741	1.44	0.301
LS-4	280	0.157	0.089	0.068
LS-5	741	0.501	0.214	0.287

**Table 3:** XPS-based quantitative functionalities C, O, and S in LS-1 to -5.

Elements (%)		LS-1		LS-2		LS-3		LS-4		LS-5	
**C (%)**	*C-C sp^2^*	77.3	87.2	69.5	79.6	73.0	80.1	39.0	51.3	58.4	69.6
	*C-C sp^3^*	9.0		8.8		6.4		9.5		9.5	
	*C-O, C-S*	0.9		1.2		0.7		2.2		1.3	
	*C=O*	0		0.0		0.8		0.6		0.5	
**S (%)**	*S-C*	1.9	3.0	3.5	6.5	0.4	1.0	6.5	12.6	5.5	8.9
	*S=C-O*	0.2		0.5		0.1		1.1		1.0	
	*SO_x_*	0.9		2.5		0.6		5.0		2.4	
**O (%)**	*S=O, C-O, OH*	6.0	8.8	9.8	11.5	8.6	13.5	26.5	30.9	8.9	11.2
	*C-O-H*	2.8		1.8		4.8		4.5		2.3	

## Data Availability

Not applicable.

## Supplementary Materials

The following supporting information can be downloaded at: https://www.mdpi.com/article/10.3390/ma16010455/s1, Table S1: Sips Constants values.
