# Supplementary material for: One-Step Synthesis of Sulfur-Doped Nanoporous Carbons from Lignin with Ultra-High Surface Area, Sulfur Content and CO2 Adsorption Capacity
Source: Materials (Basel). 2023 Jan 3;16(1):455. doi: 10.3390/ma16010455 (PMC9822399; doi:10.3390/ma16010455)
Supplement: Supplementary file 1 [file materials-16-00455-s001.zip › materials-2123349-supplementary.pdf]

Supporting Information

One-Step Synthesis of Sulfur-Doped Nanoporous Carbons from Lignin with Ultra-High Surface Area, Sulfur Content and CO_2 Adsorption Capacity

Dipendu Saha *, Gerassimos Orkoulas and Dean Bates

Chemical Engineering Department, Widener University, 1 University Place, Chester, PA 19103, USA

* Correspondence: dsaha@widener.edu, Tel.: +1-610-499-4056, Fax: +1-610-499-4059

Table S1. Sips Constants values.

**Samples**	**Constants**	**CO2**	**CH4**	**N2**
**LS-1**	am	4.33828027	1.93808298	7000
	b	0.00075371	0.00049067	1.1174E-07
	n	0.96984617	0.94422089	1.07314567
**LS-2**	am	13.2484922	244.805507	7128.80823
	b	0.00393657	9.0754E-05	1.1174E-07
	n	1.49426239	1.63931258	1.07673099
**LS-3**	am	511.635183	9.49762032	2.22610627
	b	8.4685E-05	0.00049978	0.00036948
	n	1.19472931	1.05604419	1.01687262
**LS-4**	am	4.47440344	0.88811551	0.75090376
	b	0.00232267	0.00102023	0.00019535
	n	1.30996255	1.1058805	0.985238
**LS-5**	am	2.12822949	1.49855532	0.05910709
	b	0.00021294	0.0006759	7.6156E-06
	n	0.84367693	1.08001069	0.54040259
